# Mixed Comparison of Different Exercise Interventions for Function, Respiratory, Fatigue, and Quality of Life in Adults With Amyotrophic Lateral Sclerosis: Systematic Review and Network Meta-Analysis

**DOI:** 10.3389/fnagi.2022.919059

**Published:** 2022-07-11

**Authors:** Yining Zhu, Yining Xu, Rongrong Xuan, Jialu Huang, Bíró István, Gusztáv Fekete, Yaodong Gu

**Affiliations:** ^1^Faculty of Sports Science, Ningbo University, Ningbo, China; ^2^The Affiliated Hospital of Medical School, Ningbo University, Ningbo, China; ^3^Faculty of Engineering, University of Szeged, Szeged, Hungary; ^4^Savaria Institute of Technology, Eötvös Loránd University, Szombathely, Hungary

**Keywords:** amyotrophic lateral sclerosis, exercise, prom, systematic review, network meta-analysis

## Abstract

**Background:**

Amyotrophic lateral sclerosis (ALS) is a progressive neuromuscular disease whose primary hallmark is the progressive degeneration of motor neurons in the brainstem, spinal cord, and cerebral cortex that leads to weakness, spasticity, fatigue, skeletal muscle atrophy, paralysis, and even death. Exercise, as a non-pharmacological tool, may generally improve muscle strength, cardiovascular function, and quality of life. However, there are conflicting reports about the effect of exercise training in adults with ALS.

**Aims:**

This systematic review and network meta-analysis aim to conduct a mixed comparison of different exercise interventions for function, respiratory, fatigue, and quality of life in adults with ALS.

**Methods:**

Randomized controlled trials with ALS participants were screened and included from the databases of PubMed, Medline, and Web of Science. Physical exercise interventions were reclassified into aerobic exercise, resistance training, passive exercise, expiratory muscle exercise, and standard rehabilitation. Patient-reported outcome measures would be reclassified from perspectives of function, respiratory, fatigue, and quality of life. The effect size would be transferred into the percentage change of the total score.

**Result:**

There were 10 studies included, with the agreement between authors reaching a kappa-value of 0.73. The network meta-analysis, which was conducted under the consistency model, identified that a combined program of aerobic exercise, resistance exercise, and standard rehabilitation showed the highest potential to improve quality of life (0.64 to be the best) and reduce the fatigue (0.39 to be the best) for ALS patients, while exercise program of aerobic and resistance training showed the highest potential (0.51 to be the best) to improve ALS patients' physical function. The effect of exercise on the respiratory was still unclear.

**Conclusion:**

A multi-modal exercise and rehabilitation program would be more beneficial to ALS patients. However, the safety and guide for practice remain unclear, and further high-quality randomized controlled trials (RCTs) with a larger sample are still needed.

**Systematic Review Registration:**

https://www.crd.york.ac.uk/prospero/display_record.php?ID=CRD42021253442, CRD42021253442.

## Introduction

Amyotrophic lateral sclerosis (ALS), whose primary hallmark is the progressive degeneration of motor neurons in the brainstem, spinal cord, and cerebral cortex that leads to weakness, spasticity, fatigue, skeletal muscle atrophy, paralysis, and even death, is described first in 1869 (Cleveland and Rothstein, 2001; Byrne et al., 2011). Due to muscle weakness and spasticity, which are the primary symptoms reported in patients with ALS, the functional ability has been damaged in patients with ALS (Jensen et al., 2016). Moreover, musculoskeletal dysfunction would induce respiratory dysfunction, which is the most common cause of death in ALS patients (Corcia et al., 2008).

ALS is one of the most common progressive neuromuscular diseases that is usually regarded as a disease that cannot be completely cured, and the majority of ALS patients are elderly population. The treatments of ALS are often aimed at strengthening the physical function of patients, improving their perceived feelings, reducing the incidence of adverse events, and comprehensively improving their quality of life. Currently, standard rehabilitation (SR) protocols, pathogenetic therapy, and various supportive therapies are prevalent in the clinical practice of ALS treatment. The SR of ALS is usually referred to multi-modal rehabilitation protocols that contain stretching exercises, active proprioceptive exercises, and basic active functional exercise, whereas there are two drugs approved for the pathogenetic therapy of ALS that have been identified to have effects on slowing down the progression of the disease (Beghi et al., 2004).

Physical exercise is one of the most common non-pharmacological tools that might be able to generally improve muscle strength and cardiovascular function of ALS patients and have a positive effect on their quality of life (Garber et al., 2011; Ying et al., 2021). For example, in 2018, a randomized controlled trial conducted by Clawson's team demonstrated that exercise programs contained resistance, endurance, and stretching (or range of motion) exercises are safe to be performed with the specified regimen without any worsening of outcomes as related to ALS function and can be tolerated showing high compliance over a long term (Clawson et al., 2018). A systematic review published in 2021 identified that therapeutic physical exercise could contribute to slowing down the deterioration of the musculature of patients with ALS and facilitating the performance in daily life activities in the short-, medium, and long term (Ortega-Hombrados et al., 2021). In addition, an improved overall functional score, muscle strength, and quality of life in ALS patients had been reported following a period of exercise intervention (Jensen et al., 2017). It seemed highly probable that any form of exercise has an advantageous impact on the quality of life of ALS patients (Tsitkanou et al., 2019).

However, the large heterogeneities within exercise protocols that used in clinical practice make the evidence level of exercise treatments for ALS low. For example, a randomized controlled trial conducted by Zucchi's team in 2019, which explored the effect of high-frequency motor rehabilitation for ALS patients, found that there was no significant difference between the effectiveness of high and usual frequency physical exercise on the ALS Functional Rating Scale—Revised (ALSFRS-R) scores, motor and respiratory functions, survival, fatigue, and quality of life of ALS patients. Moreover, a systematic review and meta-analysis published in 2014 demonstrated that there was limited evidence that inspiratory muscle training leads to the strengthening of inspiratory muscles in ALS. Improvements were minor in only a few parameters, but the survival time was significantly longer in the ALS patients who did inspiratory muscle training (Eidenberger and Nowotny, 2014). Another systematic review and meta-analysis conducted by Rahmati's team found that there was a statistically significant difference in favor of physical exercise in functional ability, overall quality of life, and aerobic capacity for ALS patients, but there was no significant difference in respiratory function, fatigue, pain, and body strength (Rahmati and Malakoutinia, 2021).

Additionally, the potential negative effects of physical exercise, such as accelerating disease progression or increasing adverse events described in the previous studies, might be related to the form of exercise (Mahoney et al., 2004; Harwood et al., 2009; Tsitkanou et al., 2019; Xiang et al., 2022; Xu et al., 2022). For example, a study conducted by Tsitkanou's team in 2019 found that the motor neuronal injuries of ALS patients might increase their risk of falls during physical exercises, such as aerobic exercise (AE) on a treadmill or bicycles. Moreover, a study made by Harwood's team published in 2009 claimed that the dose-response of physical exercise in healthy individuals appears to be changed in ALS patients, leading them to have a greater risk of overtraining when they conduct long-terms of resistance training (RT).

Since there were also conflicting reports about the effect of exercise training in ALS, the best way to exercise for patients with ALS has not been determined, and the effectiveness of exercise training as a treatment for other symptoms of ALS in human studies is not yet clear. For instance, the effects of respiratory training programs on ALS patients had been addressed in a previous meta-analysis (Ferreira et al., 2016). In 2013, a Cochrane review was conducted to investigate the effect of physical exercise for patients with ALS on the overall functional score, fatigue, muscle strength, and quality of life without considering the form of exercises. The review found that the results of synthesis evidence were unclear because only a few studies were included (Dal Bello-Haas and Florence, 2013).

Many more studies of different physical exercise forms for ALS patients have been conducted recently. Therefore, it was necessary to conduct an updated systematic review to clarify the effects of different physical exercise intervention programs on ALS patients in terms of overall functional score, respiratory function, perceived fatigue, and quality of life. In addition, since the existing studies usually contained different experimental groups and control groups, making it difficult to conduct a pair-wise meta-analysis that could only compare the effectiveness of two kinds of interventions at the same time, a network meta-analysis approach, which was an expansion of pair-wise meta-analysis, could be able to compare more than two interventions for a certain disease synchronously. This systematic review and network meta-analysis were aimed to conduct a mixed comparison of different exercise interventions for function, respiratory, fatigue, and quality of life in adults with ALS.

## Methods

### Program and Registration

This systematic review was written according to the Preferred Reporting Items for Systematic Reviews and Meta-Analysis guidelines (PRISMA) (Moher et al., 2009). Literature screening criteria and study search strategy were proposed and agreed upon by two independent authors (YZ and XY). The PROSPERO registration number of this systematic review and network meta-analysis was CRD42021253442.

### Eligibility Criteria

#### Population/Participants

This systematic review aimed to include all studies with adult ALS patients over 18 years old as participants in trials with the diagnosis criteria of ALS that are defined according to the revised El Escorial Criteria (Brooks et al., 2000).

#### Intervention(s)

All the interventions in this review were classified into six basic categories according to the definition in these included studies. These basic categories were AE, RT, SR, passive exercise (PE), expiratory muscle exercise (EE), and daily activity (DA). What should be emphasized was that the category SR contained active stretching exercise, active proprioceptive exercises, basic active functional exercise, or any forms of sham (or placebo) treatments, while the category PE represented movement exercise induced by special therapists (Lunetta et al., 2016). Each combined intervention program that contained two or more basic categories was represented by basic categories' abbreviations connected by plus signs, for example, “AE + RT + SR” meant a comprehensive exercise program that included cycling, strength exercises, and proprioceptive exercises. The description of interventions and comparators is provided in Table 1.

**Table 1:** Description of basic interventions/comparators.

**Interventions/comparators**	**Abbreviation**	**Description**
Aerobic exercise	AE	Exercise on a cycle ergometer, treadmill, or rowing machine under an intensity below the lactic acid threshold.
Resistance training	RT	Active muscle exercise of endurance, strength, or power against gravity, external loaded weight, or fitness equipment.
Standard rehabilitation	SR	Rehabilitation protocols that contained stretching, proprioceptive exercises, functional exercise, or any forms of sham (or placebo) treatments.
Passive exercise	PE	Movement exercises induced by special therapists
Expiratory muscle exercise	EE	Expiratory muscle strength training or breathing exercise with the help of expiratory training devices.
Daily activity	DA	Maintaining usual daily activities

#### Comparator(s)

The eligibility criteria of the comparator(s) were the same as the intervention(s). Network meta-analysis was particularly flexible for fitting complex models, including multi-arm trials, and provided credible intervals and rank probabilities for comparing the overall effectiveness of treatments based on the Bayesian approach. All the treatments were ranked by their estimated effect sizes, and then, across all samples, averages for the first rank, second rank, and so on (Lumley, 2002; Chung and Lumley, 2008; Salanti et al., 2011; Wang et al., 2012). These estimated probabilities were plotted against the ranks. Therefore, network meta-analysis was feasible to make mixed and indirect intervention comparisons (Mills et al., 2013).

#### Outcomes

In clinical practice, clinicians used the patient-reported outcome measures (PROMs) to assess the specific functional abilities and life quality of patients. There were a lot of PROMs tools whose reliability had already been verified in the assessment of ALS patients from different perspectives. The PROMs in this systematic review are listed as follows.

##### Overall Functional Score

ALSFRS was the most common outcome measure in clinical trials researching ALS patients. There were two kinds of ALSFRS commonly used in clinical practice. One was the ALSFRS-R. The ALSFRS-R included 12 questions and was rated on a 5-point Linkert scale from 0, which referred to being unable to perform the task to 4, which referred to normal ability (Cedarbaum et al., 1999). The other was the original version of ALSFRS, which had 10 questions whose scores ranged from 0 to 4. The total scores of the original version of ALSFRS were between 0, which represented the worst, and 40, which represented the best (Group, 1996).

##### Respiratory Function

The percent predicted value for forced vital capacity (FVC) was selected to represent the respiratory function of ALS patients.

##### Perceived Fatigue

The fatigue severity scale (FSS), which was a self-report scale of nine items examining motivation, physical function, responsibilities, work, family or social life, exercise, fatigue, frequency of problems, and priority of symptoms with scores of answers ranged from 1 that referred to strongly disagree to 7 that referred to strongly agree, was selected to represent the perceived fatigue of ALS patients. There was good evidence of its psychometric properties and clinical feasibility in measuring fatigue in various neurological conditions (Tyson and Brown, 2014). Moreover, the Checklist for Individual Strength-Fatigue (CIS-Fatigue), which was a 20-item self-report questionnaire that captured four domains of fatigue, including subjective experience of fatigue, reduction in motivation, reduction in activity, and reduction in concentration, was also chosen to represent the perceived fatigue of ALS patients. The CIS-Fatigue had demonstrated satisfactory psychometric properties, including high internal consistency and the ability to discriminate against healthy individuals (Dittner et al., 2004).

##### Quality of Life

Three kinds of PROMs were used in this systematic review to represent the quality of life. The first one was the Short Form (36) Health Survey (SF-36), an often used, well-researched, and self-reported health measure. The SF-36 comprised 36 questions covering 8 domains of health. Scores for the different domains were converted and pooled using a scoring key for a total score of low to high quality of life. The SF-36 had been found valid, reliable, and responsive to change across diverse clinical populations (McHorney et al., 1994). The second one was the Euro-Qol-5D (EQ-5D), which was widely used in different countries by clinical researchers in various clinical areas. EQ-5D was one of the handful of measures recommended for use in cost-effectiveness analyses and had been translated into most major languages (Rabin and de Charro, 2001). The last PROM for life quality assessment was the McGill Quality of Life (McGill-QoL) questionnaire. The McGill-QoL assessed six domains, including a single item (a single question about the overall quality of life in the past 2 days), physical symptoms or physical problems, psychological symptoms or psychological problems, physical well-being, and existential well-being, and support (Cohen et al., 1995).

#### Study Design

Only studies of randomized controlled trials were eligible for this systematic review.

#### Exclusion Criteria

Studies were excluded if: (1) The participants were under18 years old or physical disable; (2) the participants of the trial were from a mixed population with different progressive neuromuscular diseases; (3) the intervention of the trial could not be reclassified into AE, RT, SR, PE, EE, or DA; (4) the participants of the trial took supplementation, such as nitric oxide, creatine, or caffeine, that could improve respiratory function or sporting performance; (5) the participants had clinical exercise contraindication; (6) the study was a published abstract.

### Information Sources

Since the revised El Escorial Criteria of ALS diagnosis was published in December 2000, a comprehensive, reproducible search strategy was conducted on the following databases from December 2000 to February 2022: PubMed, Embase, and Web of Science. Reference lists of included studies had also been searched. Gray literature was searched to identify potential studies. The authors were contacted and requested missing data if the original data of articles were insufficient.

### Search Strategy

The search terms used in each database were set according to the following logic: (1) contained the term “ALS” or “amyotrophic lateral sclerosis” in the title; (2) contained the term “exercise,” “train,” or “training” in the title; (3) contained the term “randomized” or “randomised” in title or abstract.

### Study Selection

All potential studies were imported into EndNote X9 (Thomson Reuters, Carlsbad, California, USA) after removing duplicates. Title, abstract, and full-text screening were made by two independent authors (YZ and XY). Any disagreement was resolved by a third independent reviewer (YG).

### Data Collection Process

Data were extracted by two independent authors (YZ and XY). An independent reviewer was invited to check all the collected data (RX).

### Data Items

The following information was collected and recorded. (1) Demographic characteristics, such as mean age and gender ratio; (2) information about intervention programs, such as names of interventions, details of programs, and the categories they could be reclassified; (3) information on each outcome measure, such as the names the of the PROMs scales and their overall scores; (4) data would be used in network meta-analysis, such as sample size of every group and results of each outcome measures in each record point, as well as the number of lost to follow up. All the data is provided in the Supplementary Material.

### Risk of Bias Assessment

The Cochrane Collaboration Risk of Bias Assessment Tool was used to assess the risk of bias (Robertson et al., 2014). All the included studies were assessed by two independent authors (YZ and XY). Any disagreement would be discussed, and an independent arbitrator (YG) was invited when an agreement could not be met. Agreement between authors was determined by Cohen's Kappa value.

### Data Pre-Processing

Data pre-processing and analysis were made by one independent author (YZ) using Microsoft Office Excel (Version 16.0 Microsoft Corporation, Redmond, WA, USA). The overall scores of every PROMs scale in each included study were recorded. Since different PROMs scales had different overall scores, after combining and comparing the results of different PROMs in a unified unit, all the original data was transferred into the percentage of overall score (Mean% ± SD%) by using a mathematical conversion, as shown in Equation (1). Moreover, since some included studies only reported the scores of subscales, mean scores and their standard deviations of every subscale were converted and then pooled together by Equations (2) and (3). Additionally, some outcome measures such as the quality of life and perceived fatigue were measured by different PROMs scales, Equations (2) and (3) were also used to pool scores of different scales after these scores had been transferred into the percentage of overall scores.


(1) $$\begin{array}{l} \begin{array}{ccc} Mean\%\pm SD\% & = & \frac{Mean\;Score}{Overall\;Score}\times 100\% \end{array}\\ \;\begin{array}{cc} \pm & \frac{SD}{Overall\;Score}\times 100\% \end{array} \end{array}$$

(2) $$Mean_{total}=\sum Mean_{sub}$$

(3) $$SD_{total}=\sqrt{\sum SD_{sub}^{2}}$$

Another independent author was invited to check all the original data and their pre-processing results to ensure there was no error.

### Synthesis of Results

The Aggregate Data Drug Information System (ADDIS V1.16.8 Produced by Drugis.org, http://drugis.org/software/addis/index) was applied to pool all the processed data into network meta-analysis, calculate the effect size, and output results.

### Risk of Bias Across Studies

The Cochrane Collaboration Risk of Bias Assessment Tool was used to assess the risk of bias across the included studies (Robertson et al., 2014). The assessment was conducted according to the results of the bias risk assessment of individual studies.

### Network Meta-Analysis

#### Network Geometry

The network geometries provided key information about the strength of evidence and displayed the number and form of interventions by the Bayesian simulation modeling. Every node in the network geometry represented one intervention; the lines referred to a direct comparison between each pair of interventions, while the number of comparison arms was represented by the number on each line (Salanti et al., 2011).

#### Consistency and Inconsistency Analysis

The consistency of the evidence structure was identified at first since the network meta-analysis was based on the homogeneity, similarity, and consistency hypothesis (Song et al., 2008). If there were closed loops in the evidence structure, the evidence structure was more complex, and the network meta-analysis was called a mixed intervention comparison, and the inconsistency occurred.

There were two approaches to identify the inconsistency. First, the random-effects standard deviations were calculated under both consistency and inconsistency models to determine if there was inconsistency within interventions. If the random-effects standard deviations under the two models were identical, it meant that there was a good consistency with the interventions. Second, the *P*-values calculated in the analysis of the node splitting were checked to determine which model could be used. The node-splitting analysis is an alternative method to assess inconsistency in network meta-analysis. It assessed whether direct and indirect evidence on a split node was in agreement. The node-splitting analysis was performed within a Bayesian framework and was computationally more intensive than other approaches. Whether the identified discrepancy was statistically significant was determined by examining the calculating a respective Bayesian *P*-value. If the *P*-value of all the direct and indirect evidence comparisons were larger than 0.05, the consistency model could be used to conduct the network meta-analysis (Dias et al., 2010).

If there was no relevant inconsistency, or there was no closed loop in the evidence structure, a consistency model was used to conclude the relative effect of the included interventions (Lu and Ades, 2006), and the network meta-analysis was called an adjusted indirect intervention comparison. Under the consistency model, the results were shown in the rank probability plot (Dias et al., 2013).

#### Ranking of Measures and Probability

A ranking of measures and probability was made to facilitate simultaneous inference regarding interventions. The ranking of treatments was made according to the probability of each intervention being the most effective or the least effective with the overall sum of the percentage in each row or column being 1.00 (100%).

## Results

### Search Strategy and Information Extraction

**Figure 1:**
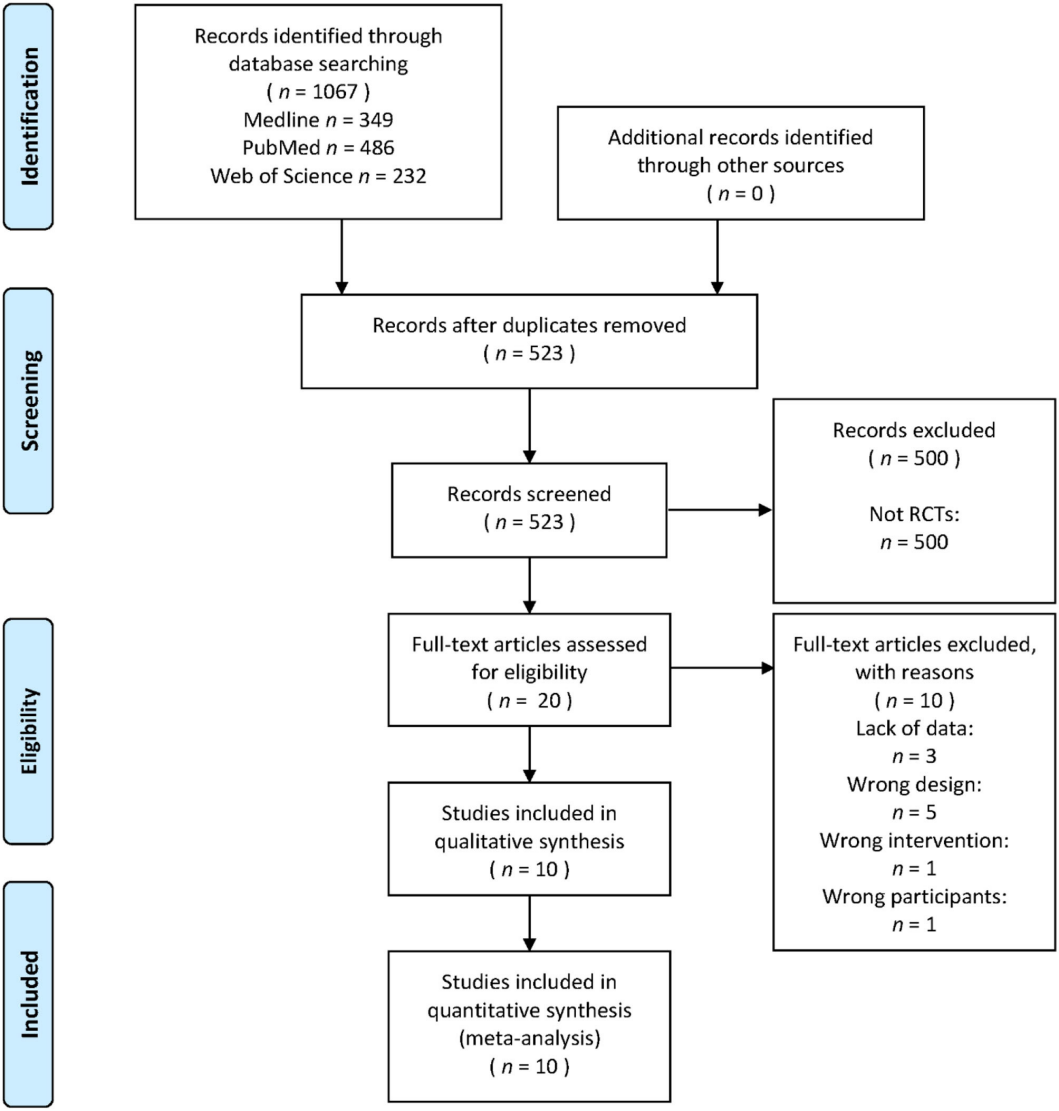
PRISMA flow diagram for the systematic review and network meta-analysis.

The search yielded 1,067 titles and abstracts for screening. After removing 544 duplicated studies, 523 studies were included in the records screening. As only randomized controlled trials (RCTs) would be included in this systematic review, 500 studies that were not RCTs were excluded, and 23 studies were included for full-text article assessment for eligibility. Among the 23 studies, three studies were excluded because of lack of data, five studies were excluded due to their ineligible design, one study was excluded because of its ineligible interventions, and one study was excluded because of its ineligible participants. Eventually, 10 studies were included in the final analysis (Drory et al., 2001; Bello-Haas et al., 2007; Pinto et al., 2012; Lunetta et al., 2016; Braga et al., 2018; Merico et al., 2018; Ferri et al., 2019; Plowman et al., 2019; van Groenestijn et al., 2019; Kalron et al., 2021). The identification process is shown as a flow diagram in Figure 1. The information of all included studies is shown in Table 2. All the original data was provided in the Supplementary Material.

**Table 2:** The information of all included studies.

**Study**	**Interventions**			**Participants**				**Outcome measures**	
	**Exercise**	**Program**	**Classification**	**Mean age**	**Number**	**Gender (F/All)**	**Lost**	**Measurement**	**Total score**
Drory et al. (2001)	Muscle endurance exercise	15-min muscle endurance exercise for limbs and trunk at home, 2/week	RT	58.0	14	6/14	6	ALSFRS FSS VAS SF-36	40 63 10 100
	Daily routine activity	Keep daily routine activity	DA	60.7	11	5/11	5		
Bello-Haas et al. (2007)	Resistance exercise	Moderate-load-and-intensity resistance exercise and stretching exercise, 3 sessions/week	RT	56.0	13	4/13	5	ALSFRS FSS FVC SF-36	40 63 100 100
	Stretching	Stretching exercise, 3 sessions/week	SR	51.8	14	7/14	4		
Lunetta et al. (2016)	Strictly monitored exercise programs (1)	Active exercise against gravity in six muscle groups in the upper and lower limbs, 3 sets of 3 reps each muscle group + 20-min cycloergometer activity for lower limbs associated with a body ergometer for upper limbs from a sitting position at 60% of maximal power output	AE + RT	61.1	30	9/30	8	ALSFRS-revised FVC McGill-QOL	48 100 140
	Strictly monitored exercise programs (2)	Active exercise program against gravity in six muscle groups in the upper and lower limbs, 3 sets of 3 reps each muscle group	RT						
	Strictly monitored exercise programs (3)	A passive exercise program consisting of 20 min of 20 flexion-extension movements per minute in 6 muscle groups in the upper and lower limbs	PE						
	Home-based passive exercise programs	Usual care program including passive exercises consisting of 20 min of 20 times flexion-extension movements/min, 6 muscle groups in the upper and lower limbs followed by stretching exercise in the four limbs, 2 days/week	PE + SR	60.3	30	13/30	5		
Braga et al. (2018)	AErobic exercise and standard care	AErobic exercise on the treadmill and standard care of daily exercises including a range of motion exercises, limbs relaxation, trunk balance, and gait training, 2 sessions/week	AE + SR	63.2	24	6/24	5	ALSFRS-revised	48
	Standard care	Standard care of daily exercises including Range of Motion exercises, limbs relaxation, trunk balance, and gait training + program at home or other rehabilitation, 2 sessions/week	SR	62.0	24	10/24	19		
Merico et al. (2018)	Specific exercise	Individualized strengthening by using rubber bands, 3 reps for bilateral muscle, and 80% max contraction + 15-20 min of aerobic endurance by using cycle ergometer or ergometry arm-leg or treadmill at 65% MHR, 7 sessions/week, 60 min	AE + RT	61.6	23	10/23	8	FIM FSS	126 63
	Standard neuromotor rehabilitation	60-min standard care including stretching exercise, active mobilization, and general muscle reinforcement, 7 sessions/week	SR	59.8	15	4/15	8		
van Groenestijn et al. (2019)	AErobic exercise	AErobic cycling exercise program on a cycle ergometer and a step board + Usual careneuropalliative care by multidisciplinary, secondary care teams, 3 sessions/week	AE + SR	60.9	27	9/27	17	ALSFRS-revised CIS-Fatigue VAS FVC SF-36	48 56 10 100 200
	Usual care	Usual care program consists of a rehabilitation medicine consultant, an occupational therapist, physical therapist, speech therapist, dietician, social worker, psychologist, and consultant physicians	SR	59.9	30	8/30	8		
Ferri et al. (2019)	Tailored exercise training	15-min cycling + 25-min strength exercises with 60 % RM + 10-min proprioceptive exercises + 10-min upper and lower body strength exercises, 3 sessions/week	AE + RT + SR	50.7	8	2/8	1	ALSFRS-revised	48
	Usual care	Maintaining usual daily activities	DA	55.5	8	2/8	4		
Pinto et al. (2012)	Respiratory exercise	Inhaling and exhaling through the Threshold-IMT device, 2 times/day, 8 months	EE	57.0	13	8/26	2	ALSFRS FIM FSS FVC EQ5D-VAS	40 126 63 100 100
	Placebo exercise	Follow a placebo exercise program for the first four months and then active exercise for the second four-month period	DA		13		4		
Plowman et al. (2019)	Expiratory Strength Training	Expiratory muscle strength training at 50% of maximum expiratory pressure, at home, 5 times/week + 1 time/week, 5 sets of 5 reps targeted forced exhalations, 8 weeks total	EE	63.1	24	7/24	1	ALSFRS FVC	40 100
	Sham expiratory Strength Training	Placebo training program with a trainer that looked identical to the high physiologic load trainer but had the internal spring removed	DA	60.1	24	12/24	1		
Kalron et al. (2021)	AErobic and strength training	2 times/week, aerobic training by recumbent cycling, flexibility achieved by stretching and passive exercises, and strength training *via* functional exercises	AE + RT + SR	60.4	16	6/16	2	ALSFRS-revised FSS FVC SF-36	48 63 100 800
	Stretching exercise	Basic stretching exercises of the upper and lower limb at home	SR	56.7	16	5/16	2		

### Risk of Bias Assessment

**Figure 2:**
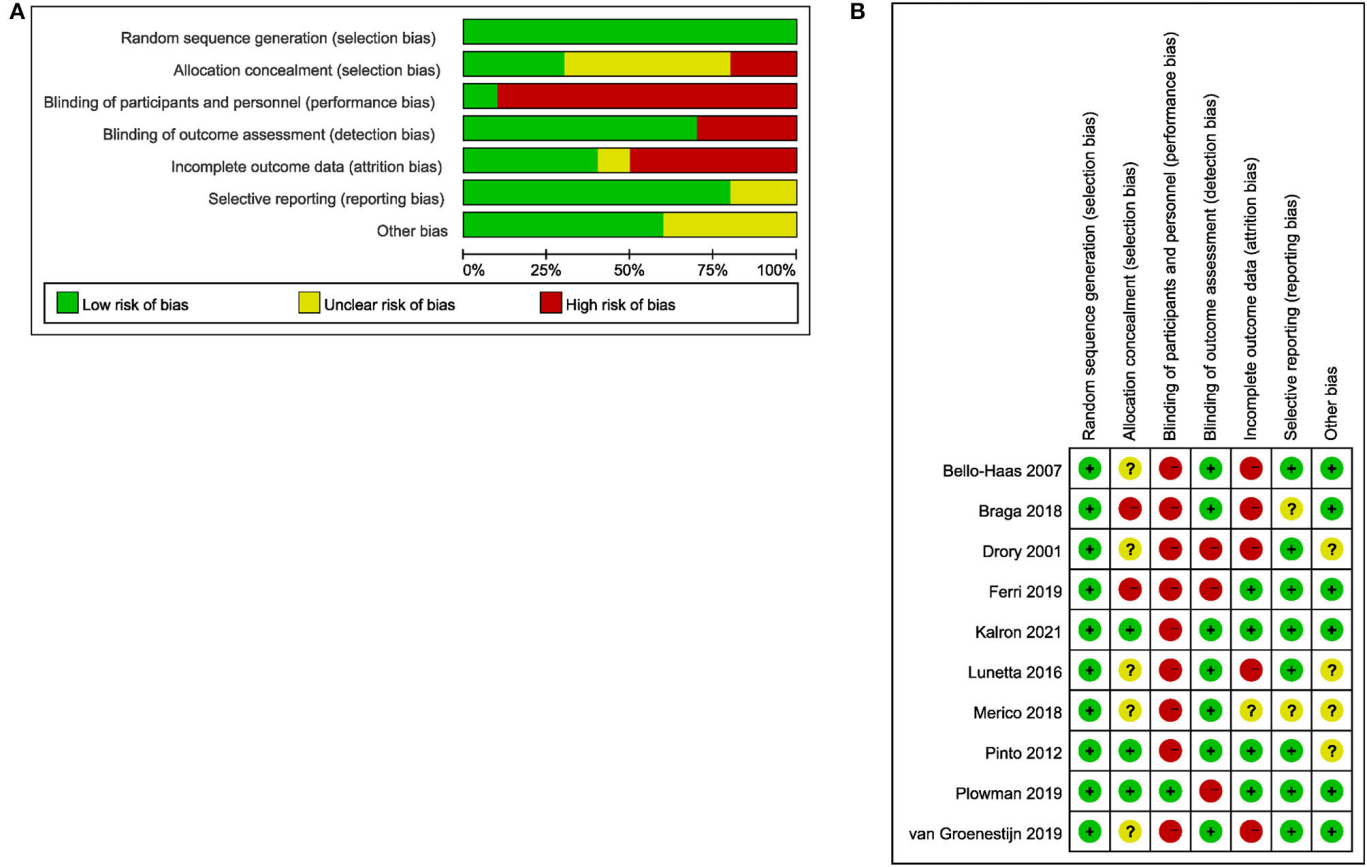
The result of the risk of bias assessment. **(A)** Risk of bias graph; **(B)** Risk of bias summary.

Figure 2 shows the assessment results of the risk of bias. According to the overall bias provided in Figure 2A: (1) the risk of performance bias (blinding of participants and personnel) was high (high in 9 studies); (2) the risk of detection bias (blinding of outcome assessors) was moderate (high in 3 studies); (3) the risk of attrition bias (incomplete outcome data) was moderate (high in 4 studies); (4) the risk of selection bias (random sequence generation and allocation concealment) was low (low in all studies); (5) the risk of reporting bias (selective reporting of outcomes) was low (low in 8 studies). According to the Cochrane Collaboration Risk of Bias Assessment Tool, a study with three or more items in high risk would be regarded as having a high risk of bias, a study with five or more items with low risk and 1 item with high risk at most would be regarded as having a low risk of bias, and a study under other conditions would be regarded as having a moderate risk of bias. It was shown in Figure 2B that two studies had a high risk of bias, five studies had a moderate risk of bias, and three studies had a low risk of bias. The agreement between authors reached a kappa-value of 0.73.

### Network Meta-Analysis

**Figure 3:**
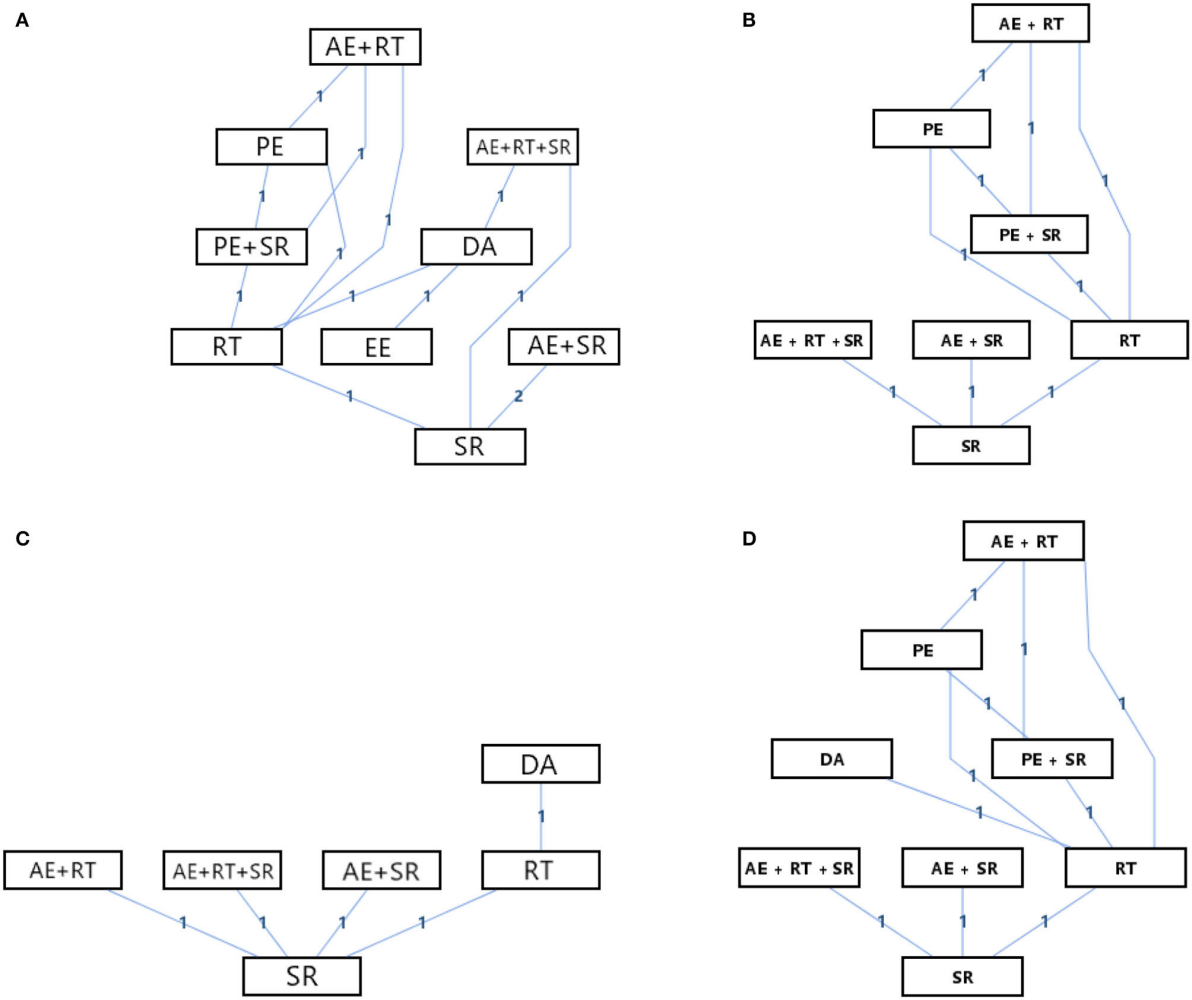
Network geometry of the mixed comparison. **(A)** Overall functional score; **(B)** Respiratory function; **(C)** Perceived fatigue; **(D)** Quality of life.

The evidence structures of the mixed interventions comparisons for the overall functional score, respiratory function, perceived fatigue, and quality of life are presented in Figure 3, while the results of the consistency and inconsistency analysis are provided in Table 3.

**Table 3:** The results of the consistency and inconsistency analysis.

**Outcome measures**	**Interventions**	**Random-effects standard deviations**		**Node-splitting analysis**	
		**Consistency model Mean (95%CI)**	**Inconsistency model Mean (95%CI)**	**Comparison**	***P*-value**
Overall functional score	AE + RT, AE + RT + SR, AE + SR, DA, EE, PE, PE + SR, RT, SR	7.99 (0.57, 22.63)	7.01 (0.24, 22.35)	AE + RT + SR vs. DA AE + RT + SR vs. SR DA vs. RT RT vs. SR	0.31 0.30 0.36 0.31
Respiratory function	AE + RT, AE + RT + SR, AE + SR. PE, PE + SR, RT, SR	4.77 (0.42, 9.07)	4.75 (0.29, 9.08)	None	
Perceived fatigue	AE + RT, AR + RT + SR, AE + SR, DA, RT, SR	10.36 (0.47, 20.52)	10.61 (0.68, 20.57)	None	
Quality of life	AE + RT, AE + RT + SR, AE + SR, DA, PE, PE + SR, RT, SR	5.54 (0.46, 10.59)	5.23 (0.03, 10.60)	None	

**Figure 4:**
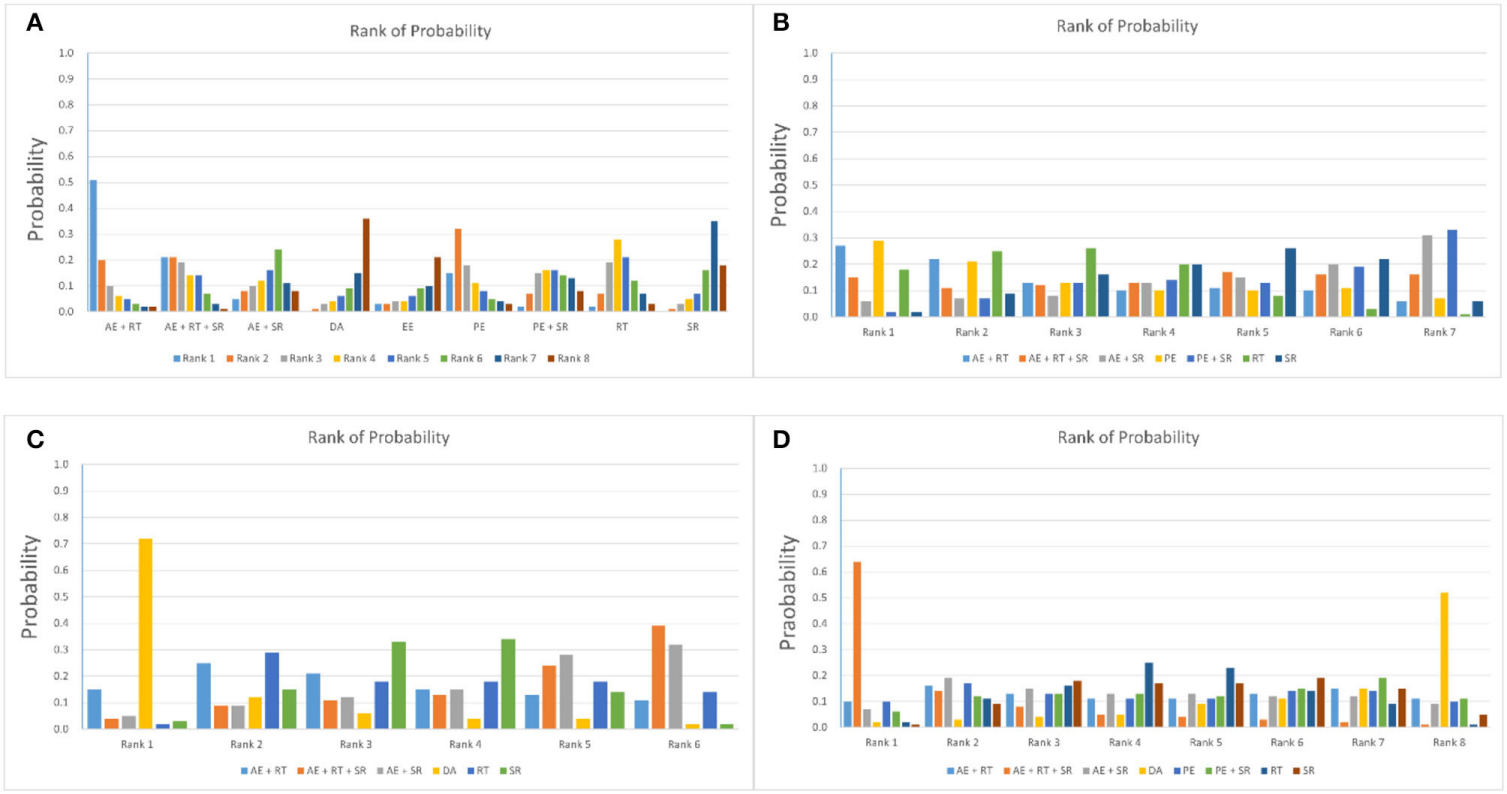
Network geometry of the mixed comparison. **(A)** Overall functional score; **(B)** Respiratory function; **(C)** Perceived fatigue; **(D)** Quality of life.

According to Figure 3, there were nine kinds of interventions included in the network meta-analysis of the effects on overall functions scores with an evidence structure of a mixed comparison; there were seven kinds of interventions included in the network meta-analysis of the effects on respiratory function with an evidence structure of a mixed comparison; there were six kinds of interventions included in the network meta-analysis of the effects on perceived fatigue with an evidence structure of an adjusted indirect comparison; and there were eight kinds of interventions included in the network meta-analysis of the effects on quality of life with an evidence structure of a mixed comparison. Besides, except for the direct comparison of SR and AE + SR, which had two arms, other direct comparisons had only one arm. According to Table 3, all the evidence of structures showed good consistency with the random-effects standard deviations calculated under consistency and inconsistency models were fully identical, and the *P*-value of all the direct and indirect evidence comparisons in the node-splitting analysis was more than 0.05. Therefore, the consistency model could be applied to conduct the network meta-analysis of all the outcomes. Figure 4 and Table 4 provide the probability rank of every intervention in each mixed comparison. It should be noted that the PROMs of the overall functional score, respiratory function, and the quality of life were continuous data that the higher would be better, while the PROMs of perceived fatigue were continuous data that the lower would be better. Therefore, in the probability rank of perceived fatigue, Rank N would be the best, whereas, in the other three PROMs domains, Rank 1 would be the best. The league table of the network meta-analysis for each outcome is provided in the Supplementary Material, the numbers showing the synthesized effect size differences of the comparison between every two interventions.

**Table 4:** The rank probability rank of each mixed interventions comparison.

**Outcome measures**	**Intervention**	**Rank 1**	**Rank 2**	**Rank 3**	**Rank 4**	**Rank 5**	**Rank 6**	**Rank 7**	**Rank 8**	**Rank 9**
Overall functional score	AE + RT	0.51	0.20	0.10	0.06	0.05	0.03	0.02	0.02	0.01
	AE + RT + SR	0.21	0.21	0.19	0.14	0.14	0.07	0.03	0.01	0.01
	AE + SR	0.05	0.08	0.10	0.12	0.16	0.24	0.11	0.08	0.06
	DA	0.00	0.01	0.03	0.04	0.06	0.09	0.15	0.36	0.26
	EE	0.03	0.03	0.04	0.04	0.06	0.09	0.10	0.21	0.40
	PE	0.15	0.32	0.18	0.11	0.08	0.05	0.04	0.03	0.02
	PE + SR	0.02	0.07	0.15	0.16	0.16	0.14	0.13	0.08	0.10
	RT	0.02	0.07	0.19	0.28	0.21	0.12	0.07	0.03	0.01
	SR	0.00	0.01	0.03	0.05	0.07	0.16	0.35	0.18	0.14
Respiratory function	AE + RT	0.27	0.22	0.13	0.10	0.11	0.10	0.06		
	AE + RT + SR	0.15	0.11	0.12	0.13	0.17	0.16	0.16		
	AE + SR	0.06	0.07	0.08	0.13	0.15	0.20	0.31		
	PE	0.29	0.21	0.13	0.1	0.10	0.11	0.07		
	PE + SR	0.02	0.07	0.13	0.14	0.13	0.19	0.33		
	RT	0.18	0.25	0.26	0.20	0.08	0.03	0.01		
	SR	0.02	0.09	0.16	0.20	0.26	0.22	0.06		
Perceived fatigue	AE + RT	0.15	0.25	0.21	0.15	0.13	0.11			
	AE + RT + SR	0.04	0.09	0.11	0.13	0.24	0.39			
	AE + SR	0.05	0.09	0.12	0.15	0.28	0.32			
	DA	0.72	0.12	0.06	0.04	0.04	0.02			
	RT	0.02	0.29	0.18	0.18	0.18	0.14			
	SR	0.03	0.15	0.33	0.34	0.14	0.02			
Quality of life	AE + RT	0.10	0.16	0.13	0.11	0.11	0.13	0.15	0.11	
	AE + RT + SR	0.64	0.14	0.08	0.05	0.04	0.03	0.02	0.01	
	AE + SR	0.07	0.19	0.15	0.13	0.13	0.12	0.12	0.09	
	DA	0.02	0.03	0.04	0.05	0.09	0.11	0.15	0.52	
	PE	0.10	0.17	0.13	0.11	0.11	0.14	0.14	0.10	
	PE + SR	0.06	0.12	0.13	0.13	0.12	0.15	0.19	0.11	
	RT	0.02	0.11	0.16	0.25	0.23	0.14	0.09	0.01	
	SR	0.01	0.09	0.18	0.17	0.17	0.19	0.15	0.05	

According to the rank of probability presented in Table 4, a combination program of aerobic and resistance exercise had the most potential to be the best intervention choice in improving the overall functional score for ALS patients (0.51 in Rank 1 and 0.01 in Rank 9), while maintaining DA, SR protocols, and EE might have the lowest probabilities to become the best intervention choices for the overall functional score of ALS patients (0.00, 0.00, and 0.03 in Rank 1 and 0.26, 0.14, and 0.40 in Rank 9). When it came to the respiratory function, there were two intervention programs with high potential, one of which was combination programs of AE and RT (0.27 in Rank 1 and 0.06 in Rank 7), and the other was PE (0.29 in Rank 1 and 0.07 in Rank 7). In contrast, programs included AE and SR programs and programs contained PE and SR showed the lowest probability to induce the best positive effectiveness (0.06 and 0.02 in Rank1 and 0.31 and 0.33 in Rank 7). A multi-model intervention program contained AE, RT, and SR, or a program that only contained AE and SR showed the highest potential to bring the largest effect on reducing perceived fatigue for ALS patients (0.39 and 0.32 in Rank 6, 0.04 and 0.05 in Rank 1), while keeping DA showed the lowest potential from this perspective (0.72 in Rank 1 and 0.02 in Rank 6). Additionally, the low potential for creating the best effectiveness of maintaining DA also occurred when it came to improving the life quality for ALS patients (0.52 in Rank 8 and 0.02 in Rank 1), whereas multi-model intervention programs contained AE, RT, and SR seemed to have the highest potential to be the best intervention from this perspective (0.64 in Rank 1 and 0.01 in Rank 8).

## Discussion

The objective of this systematic review and network meta-analysis is to conduct a mixed comparison of different exercise interventions for function, respiratory, fatigue, and quality of life in adults with ALS by using the percentage change of PROMs as outcomes. The main findings of this review are as follows. First, for ALS patients, a combined program of AE, resistance exercise, and SR might be the best intervention to improve their quality of life and reduce their perceived fatigue, while exercise programs of aerobic and RT showed the highest potential to improve their overall physical function. Moreover, only maintaining DAs might not be beneficial to ALS patients in relieving perceived fatigue or improving their quality of life. Last, the effect of exercise on the respiratory was still unclear. The main findings indicated that ALS patients could acquire the benefit induced by different exercise interventions without significant interference effect, and the positive effect induced by unimodal interventions might be weaker than multi-modal interventions.

The positive effect of multi-modal exercise interventions that contain AE, RT, and SR programs in improving the quality of life and reducing the perceived fatigue of ALS patients has already been identified by many previous studies and systematic reviews. For example, a preliminary study published in 2010 determined the feasibility, tolerability, safety, and effect size of repetitive rhythmic exercise for ALS patients, concluding that repetitive rhythmic exercise that contained both resistance and stretching movements was feasible, tolerated, and safe and was consistent with improved work capacity and gait function in patients with ALS who are dependent on assistive devices for ambulation (Sanjak et al., 2010). A systematic review published in 2021 demonstrated that physical exercise would be an efficacious and safe therapeutic intervention with a medium-sized effect on the quality of life, a large effect on mood, and a small but significant effect on improving cognition in patients with chronic brain disorders. The effect had a positive dose-response correlation, no matter the exercise was AE, resistance exercise, or concurrent exercise of endurance training and strength training (Dauwan et al., 2021).

The possible mechanism of the multi-modal exercise interventions might come from the following aspects. On one hand, AE training could reduce psychosocial stress, which is an independent risk factor for mortality in patients with chronic diseases (Milani and Lavie, 2009). On the other hand, RT could be an important factor in enhancing the quality of life and survival of patients with chronic diseases by enhancing baroreflex sensitivity by an angiotensin II-dependent mechanism induced by the mechanical tension during training (Mousa et al., 2008). Some animal experiments have also explored the physiological and psychological mechanisms of the multi-modal exercise program, an animal trial with rats as subjects explored the curative effect and possible mechanism by observing the changes in behavior, inflammatory factors, and intestinal flora in rats after exercise, claiming that the mechanism of exercise training to improve the depressive behavior of rats might be related to inhibiting the expression of proinflammatory factors and increasing the number of lactic acid bacteria in the intestine (Yang et al., 2021). When it comes to stretching exercises, stretching exercises could improve flexibility, maintain the extensibility of muscle and soft tissue, improve joint mobility, and prevent contractures. As patients with ALS usually have weaker muscles, they easily get an imbalance between agonist and antagonist muscle groups, predisposing themselves to muscle shortening, joint contractures, and poor posture such as Claw hand deformity. Stretching weakened and unaffected muscle groups could prevent contractures, maintain good alignment of body segments, reduce pain from hypomobility, and help lessen the potential complexities of functional mobility and performing activities of daily living (White and Dressendorfer, 2004).

Besides, the results of this network meta-analysis also demonstrated that exercise programs of aerobic and RT showed the highest potential to improve their overall physical function. This result is correspondent with that of some previous studies, a preliminary study conducted in 2018 demonstrated that structured home-based exercises without supervision by a physical therapist could be used to alleviate functional deterioration in patients with early-stage ALS (Kitano et al., 2018). In 2019, Sivaramakrishnan's team investigated the safety and feasibility of slowing disease progression in ALS and possibly facilitating neuroplasticity, and identified that the effect of a recumbent stepping program for improving the score of ALSFRS was similar to that of common exercise modalities, such as the treadmill and cycle ergometer (Sivaramakrishnan and Madhavan, 2019). Moreover, a narrative review published in 2008 identified that based on human and animal studies of exercise and motor neuron degeneration, it could be concluded that exercise, particularly individualized, monitored, and progressive resistance exercise, was likely to be more beneficial than deleterious for patients with ALS by inducing functional improvement (Chen et al., 2008).

This phenomenon also occurs in many previous studies that explored the effect of aerobic and resistance exercise programs on patients with other progressive neuromuscular diseases. For example, a systematic review published in 2021 claimed that cardiopulmonary exercise has a vast potential for patients with neuromuscular diseases (Barroso de Queiroz Davoli et al., 2021). Moreover, a randomized controlled trial conducted by Holm's team in 2021 identified the positive effect of strength training in addition to neuromuscular exercise and education in neuromuscular disease patients with a dose- and type-specific relationship with their pain sensitization (Holm et al., 2021). Furthermore, another systematic review and meta-analysis published in January 2022 summarized the evidence on the efficacy of AE on aerobic capacity in slowly progressive neuromuscular diseases and found that AE would be safe and leads to moderate improvement of aerobic capacity directly post-intervention in slowly progressive neuromuscular diseases, but the long-term efficacy remained unclear (Oorschot et al., 2022).

The mechanism of these results could be explained by molecular biomechanics. The molecular biomechanical generation of progressive neuromuscular diseases might be from the aggregation and folding of isomers of protein molecules in the brain (Aguzzi and O'Connor, 2010). Physical exercise could directly affect the secretion of neurotransmitters in the brain. For example, Santos-Galduroz's team found that AE under high intensity could induce the increase of neurotrophic factor secretion in the brain, promoting the formation of synapses and neurons, which is closely related to reducing the damage of progressive neuromuscular diseases to the brain of patients (Coelho et al., 2014). A trial of mice models also identified that physical exercise could delay the degeneration of central nervous system function, significantly improving the pathological characteristics of beta-amyloid protein and inhibiting the reduction of synaptic proteins as well as neurotrophic factors in the hippocampus and cerebral cortex neurons (Cho et al., 2015). And a systematic review and meta-analysis conducted in 2019 found a significant effect of AE on interleukin-6 and tumor necrosis factor-alpha decrease and positive effects on brain-derived neurotrophic factor expression (Stigger et al., 2019). Moreover, a preliminary study conducted in 2019, in which the researchers correlated clinical scales with molecular data on miRNAs (markers of myogenesis during muscle regeneration and contribute to neuromuscular junction stabilization or sprouting), identified that moderate AE could reduce miRNAs in serum, indicating that circulating miRNAs changed during skeletal muscle recovery in response to physical rehabilitation in ALS (Pegoraro et al., 2019).

The low ranking of maintaining DAs among all interventions is not surprising. Within all these categories of interventions, DA is not a treatment for ALS and is usually set as an intervention in control groups. Besides, this result could indicate that ALS might not be a self-limiting disease whose symptoms would not resolve over time. It means that if patients with ALS do not carry out a particular treatment, only keeping their DAs might not bring any positive effects for them.

What should be paid attention to was that the probability of being the best would not eliminate the uncertainty in the relative intervention effects and could spuriously give higher ranks to interventions for which little evidence is available. The probability of being the best had the disadvantage that it would not reflect the spread of rankings for the treatments, and to consider just the crude figures may be misleading. Therefore, ranking interventions based solely on the probability of each intervention being the best should be avoided (Salanti et al., 2011). At the same time, although there is no serious adverse event reported in the included studies, the safety of exercise interventions should still be worthy of attention. According to what has been mentioned in the introduction of this review, the motor neuronal injuries and induced ALS might increase the risk of falls during physical exercise, and the change in dose-response of exercise might also increase the risk of overtraining during long-term physical exercises. There are also some limitations of this systematic review. Although there was a meta-analysis published in 2020 claimed that therapeutic exercise appeared beneficial for patients with ALS and exerted more of a cardiopulmonary benefit, as opposed to preventing the progression of limb weakness (Park et al., 2020), the primary limitation is that the EE could not be included in the mix comparison for the respiratory function of ALS patients since there was not any direct comparison between EE and any intervention in the evidence structure of network meta-analysis for respiratory function. According to the specificity principle of training, it could be possible that the effect of EE on respiratory function for ALS patients would be better than other exercise programs since the EE is training specifically designed to improve respiratory function. Further studies of high evidence level clinical trials should be conducted. Another limitation of this systematic review is that, as the weight distribution in network meta-analysis is based on the arms of each direct comparison, rather than the sample size of the included trials, in this network meta-analysis, many direct comparisons have only one arm. At last, in this systematic review, the included studies only referred to the short-term effects assessed immediately after the rehabilitation. Considering the different effects of exercises that are assessed in ALS stages with different severities of symptoms, there is still a lack of knowledge of physical exercise effects on the disease progression and survival. Therefore, the weight distribution of this network meta-analysis might be too average, so the results need to be interpreted with caution.

## Conclusion

A multi-modal exercise and rehabilitation program that contains aerobic, resistance, and stretching elements would be more beneficial to ALS patients. However, the safety and guide for practice remain unclear, and randomized controlled trials with a larger sample and higher quality are still needed in the future.

## Data Availability Statement

The original contributions presented in the study are included in the article/Supplementary Material, further inquiries can be directed to the corresponding author/s.

## Author Contributions

YZ and YG: conceptualization and writing–review and editing. YX, RX, BI, and YG: methodology. YZ, YX, JH, and GF: formal analysis. YZ, YX, and RX: investigation. YZ and YX: data curation. BI, GF, and YG: supervision. All authors have read and agreed to the published version of the manuscript.

## Funding

This study was sponsored by the Ningbo Public Welfare Science and Technology Plan Project (No.2019C50095), the Health Youth Technical Talent Cultivation Special Fund Project (2020SWSQNGG-01), the Ningbo Medical Science and Technology Plan (2020Y14), the Young Cultivation Fund Project of The Affiliated of School of Medicine of Ningbo University (FYQM-KY-202003), the National Social Science Foundation of China (19ZDA352), the Key R&D Program of Zhejiang Province China (2021C03130), the Zhejiang Province Science Fund for Distinguished Young Scholars (R22A021199), and the K.C. Wong Magna Fund in Ningbo University.

## Conflict of Interest

The authors declare that the research was conducted in the absence of any commercial or financial relationships that could be construed as a potential conflict of interest.

## Publisher's Note

All claims expressed in this article are solely those of the authors and do not necessarily represent those of their affiliated organizations, or those of the publisher, the editors and the reviewers. Any product that may be evaluated in this article, or claim that may be made by its manufacturer, is not guaranteed or endorsed by the publisher.
